# A molecular framework for *lc* controlled locule development of the floral meristem in tomato

**DOI:** 10.3389/fpls.2023.1249760

**Published:** 2023-08-23

**Authors:** Hengzuo Xiang, Sida Meng, Yunzhu Ye, Leilei Han, Yi He, Yiqing Cui, Changhua Tan, Jian Ma, Mingfang Qi, Tianlai Li

**Affiliations:** ^1^ College of Horticulture, Shenyang Agricultural University, Shenyang, China; ^2^ National & Local Joint Engineering Research Center of Northern Horticultural Facilities Design & Application Technology (Liaoning), Shenyang, China; ^3^ Key Laboratory of Protected Horticulture (Shenyang Agricultural University), Ministry of Education, Shenyang, China; ^4^ Key Laboratory of Horticultural Equipment, Ministry of Agriculture and Rural, Affairs P. R. China, Shenyang, China; ^5^ Collaborative Innovation Center of Protected Vegetable Provincial Co-construction Surrounds Bohai Gulf Region, Shenyang, China

**Keywords:** tomato, *lc*, locule number, *SlWUS*, SlSEP3 transcription factor

## Abstract

Malformed tomato fruit with multiple locules is a common physiological disorder that significantly affects the quality of tomatoes. Research has shown that the occurrence of malformed fruit in tomatoes is closely linked to the number of locules, and two key QTLs, *lc* and *fas*, are involved in controlling this trait. It has been observed that *lc* has a relatively weaker effect on increasing locule number, which is associated with two SNPs in the CArG repressor element downstream of the *SlWUS*. However, the precise molecular mechanism underlying *lc* is not yet fully understood. In this study, we investigated the role of *lc* in tomato locule development. We found that the number of floral organs and fruit locules significantly increased in tomato *lc* knockout mutants. Additionally, these mutants showed higher expression levels of the *SlWUS* during carpel formation. Through cDNA library construction and yeast one-hybrid screening, we identified the MADS-box transcription factor SlSEP3, which was found to bind to *lc*. Furthermore, we observed an increase in floral organs and fruit locules similar to the *lc* ^CR^ plant on *SlSEP3* silencing plants. However, it should be noted that the *lc* site is located after the 3′ untranslated region (UTR) of *SlWUS* in the tomato genome. As a result, SlSEP3 may not be able to exert regulatory functions on the promoter of the gene like other transcription factors. In the yeast two-hybrid assay, we found that several histone deacetylases (SlHDA1, SlHDA3, SlHDA4, SlHDA5, SlHDA6, SlHDA7, and SlHDA8) can interact with SlSEP3. This indicated that SlSEP3 can recruit these proteins to repress nucleosome relaxation, thereby inhibiting *SlWUS* transcription and affecting the number of locules in tomato fruit. Therefore, our findings reveal a new mechanism for *lc* playing a significant role in the genetic pathway regulating tomato locule development.

## Introduction

Fruit size and shape are crucial agronomic traits that have greatly influenced the domestication and improvement of tomato varieties. Compared with wild tomatoes, such as *Solanum pimpinellifolium*, the number of floral organs and fruit locules in cultivated tomatoes (*Solanum lycopersicum*) increases by two to five times, or even more, which directly results in the incidence of malformed fruits with multiple locules increasing with fruit size (Lippman and Tanksley, 2001; Tanksley, 2004). In addition to environmental factors such as light, water, and nutrients, the occurrence of malformed fruit is also affected by genetic factors. Fasciated (*fas*) and locule number (*lc*) are two key QTLs that control tomato locule number and fruit size (Tanksley, 2004; van der Knaap et al., 2014). The development of tomato locules is closely associated with the differentiation of stem cells in the shoot apical meristem (SAM) and floral meristem (FM).

The mutation of *CLAVATA3* (*CLV3*) and its ligands in the CLV-WUS pathway can lead to abnormal plant development in *Arabidopsis* and tomatoes. These mutations result in excessive proliferation of stem cells, expansion of the meristem, and the production of additional organs such as flowers and fruits (Xu et al., 2015; Somssich et al., 2016). *SlCLV3*, which is identified as a candidate gene for *fas*, regulates the differentiation of tomato stem cells through the CLV-WUS pathway (Somssich et al., 2016; Chu et al., 2019). The phenotype of the *slclv3* mutant is similar to that of *fas*, but most *slclv3^pro^* lines after editing the *cis*-regulatory elements (CRE) of the *SlCLV3* promoter show more locules than *fas*, with some lines reaching 15–16 locules (Wang et al., 2021). In the absence of *SlCLV3*, *SlCLE9* initiates a weak and partial compensatory effect. The phenotype of *slcle9* is similar to the wild type, but the *slcle9*-*slclv3* double-mutant shows a more severe phenotype than the *slclv3* mutant (Rodríguez-Leal et al., 2019).

The inversion at *fas* results in a partial loss of function of *SlCLV3*, while two SNP mutations at *lc* lead to a gain of function of *SlWUS* (Huang and van der Knaap, 2011; Xu et al., 2015). Compared with *fas*, *lc* has a weaker effect on increasing locule number, which is associated with two SNPs in the CArG repressor element (similar to CRE) downstream of *SlWUS* (Muños et al., 2011; Somssich et al., 2016). *SlWUS* plays a direct role in regulating locule numbers. Its expression is increased after mutation of the CArG repressor element, suggesting that *lc* affects tomato locule number through *SlWUS* (Li et al., 2017). *SlWUS* is highly expressed in shoot apical and young flower buds, and gene silencing results in significantly smaller flowers and fruits with fewer locules (van der Knaap et al., 2014; Li et al., 2017). Furthermore, the regulatory factors in the plant hormone pathway that interact with *SlWUS* can also have a significant impact on tomato locule number (Su et al., 2022). In homozygous *slwus* mutants, SAM activity is terminated, and the transition to reproductive growth is disrupted, resulting in the absence of locules. Interestingly, when the CREs of the *SlWUS* promoter are edited, the floral organs and fruit shape of *slwus^pro^* mutants develop normally. However, only the *slwus^pro-6^* mutant, which carries an inserted 223-bp fragment and an inverted 554-bp fragment of CRE, shows a phenotype similar to *lc* (Wang et al., 2021). This suggests that the role of *lc* in regulating tomato locule number is primarily mediated through *WUS* in the CLV-WUS regulatory module. However, it remains unknown whether *lc* has other regulatory pathways besides *SlWUS* through CRE elements.

In summary, the occurrence of malformed tomato fruit has a significant impact on yield and quality, which are primarily closely related to locule number. The regulation of locule number in tomatoes is influenced by two key factors: *lc* and *fas*. While the role of *fas* in this process has been well-established through the CLV-WUS module, the mechanism of how *lc* participates in tomato locule numbers remains unclear. In this study, we utilized CRISPR/Cas9 technology to generate a tomato *lc* knockout mutant. The homozygous mutant, which had a normal *fas* and a mutated *lc*, exhibited an increase in floral organs and fruit locules. Additionally, *SlWUS* expression, which is known to be associated with locule development, was significantly increased in the CR-*lc*#11 mutant. It indicated that *lc* affected *SlWUS* by some unknown pathway. Therefore, exploring the regulatory network between *lc* and *SlWUS* will further improve the molecular mechanism of tomato locule development.

## Materials and methods

### Plant material and sample extraction

Tomato AC (*S. lycopersicum* cultivar “Ailsa Craig”) with two locules was used as material for constructing *lc* ^CR^ mutants, *lc* ^CR^ T_1_ generation was grown in a greenhouse (16 h of light at 25°C, 8 h of dark at 15°C), and conventional cultivation management was carried out.

Young leaves from the *lc* ^CR^ T_1_ generation were sampled for target detection. The shoot apicals from both *lc* ^CR^ T_1_ generation and AC were taken during the carpellary formation stage for qRT-PCR analysis; three biological replicates were set up for each sample. The shoot apical from AC were taken at different stages of flower bud differentiation, including early flower bud differentiation (S_1_), middle flower bud differentiation (sepal and petal formation stage (S_2_)), and late flower bud differentiation stage (carpel formation stage (S_3_)). Every 15–20 shoot apical samples were collected for library construction and qRT-PCR analysis. Both samples were stored in an ultra-low-temperature refrigerator to be determined.

### Vector construction and plant transformation

One single guide RNA (sgRNA) was designed to target *lc* for obtaining genome-edited mutants. The sgRNA sequence is listed in the **Supplementary Table**. The U6-*lc*-sgRNA was cloned into CRISPR/Cas9 binary vector pCBC-DT1T2-tomatoU6 to generate pCBC-DT1T2-tomatoU6-*lc*. This construct was then transformed into competent cells of *Agrobacterium* (*Agrobacterium tumefaciens*) strain LBA4404 using electroporation. Positive colonies obtained were used to transform the wild-type tomato AC through leaf disc cocultivation. Each primary transformant (*T*_0_) was genotyped by sequencing PCR products amplified from the relevant target region.

### PCR identification

The plant genomic DNA extraction kit (Tiangen, Beijing, China) was used to extract genomic DNA, following the manufacturer’s instructions (Code No. DP305, Tiangen). The extracted products were sequenced and compared with Cas9/gRNA using DNAMAN9.0 to determine whether the target was mutated and the specific mutation mode. Primers used are listed in the **Supplementary Table**.

### Phenotypic analysis

*lc* ^CR^ *T*_1_ generation mutants and wild-type plants were selected for 10 strains, respectively, and the number of sepals, stamens, petals, and locules in the first inflorescence flowers and fruit were investigated. Additionally, the number of sepals, petals, and fruit locules of flowers in the first and second inflorescence for *SlSEP3* silenced strains and wild-type plants were investigated. Data statistics and significant differences were calculated and analyzed by Excel software.

### RNA extraction and qRT-PCR analysis

Total RNA from shoot apical of *lc* ^CR^ mutants and AC was extracted using TRIzol reagent (Invitrogen, Carlsbad, California, USA). The cDNA was synthesized using PrimeScript RT Master Mix (TaKaRa, Kusatsu, Shiga, Japan) and diluted fourfold in ddH_2_O. Gene-specific primers were designed using the National Center for Biotechnology Information (NCBI) website (https://www.ncbi.nlm.nih.gov/tools/primer-blast/index.cgi). Real-time quantitative analysis was performed using TB Green^®^ Premix Ex Taq II (TaKaRa, Japan). The 20-μL reaction system contained 2 μL of diluted cDNA, 10 µL of 2 × TB Green Premix Ex Taq, and 0.3 μM of each primer with a qTOWER3/G real-time system (Analytik Jena, Jena, Thuringia, Germany). The reaction program consisted of 95°C for 30 s, 95°C for 5 s (45 cycles), 60°C for 34 s (45 cycles), and Melt for 15 s. The tomato *actin* (NCBI: NM_001330119.1) was used as an internal control. Primers are listed in the **Supplementary Table**.

### cDNA library construction

Total RNA was extracted from shoot apicals at different stages of flower bud differentiation using TRIzol reagent (Invitrogen, USA). The extracted RNA was then evaluated for integrity, purity, and concentration. An equal amount of RNA from each stage was mixed, and 1 μg was used as a template. The first strand of cDNA was synthesized using SMART cDNA Library Construction Kit (Taraka, Japan) according to the manufacturer’s instructions (Code No. 634901, Taraka). The second strand cDNA was amplified using Advantage 2 PCR Kit (Taraka, Japan) following the manufacturer’s instructions (Code No. 639206, Taraka). Double-stranded cDNA was purified using the DNA Fragment Kit (Taraka, Japan) according to the manufacturer’s instructions (Code No. 9761, Taraka), and *Sfi*I was used for enzyme digestion. Short fragments were purified using CHROMA SPIN-1000-TE (Taraka, Japan) according to the manufacturer’s instructions (Code No. 636079, Taraka). The cDNA sample with the removed short fragment was attached to three pGADT7-*Sfi*I containing different reading frames using a DNA ligation Kit (Taraka, Japan) according to the manufacturer’s instructions (Code No. 6022, Taraka). Three primary cDNA libraries were transformed into *Escherichia coli* HST08 premium electro-cells by electric shock according to the manufacturer’s instructions (Code No. 9028, Taraka). The capacity of the library was calculated by diluting the transformed products 10^3^-fold and culturing them on an LB agar medium. The number of single colonies obtained was multiplied by dilution times and the total volume of solution (mL) and then divided by the volume of culture medium (mL) to calculate the capacity of the library. To use 2×Taq PCR Mastermix (Tiangen, Beijing) for PCR amplification, 48 single colonies were randomly selected. The primary cDNA library was transformed into HST08 again, and all single colonies were collected for germiculture. The Plasmid Purification Kit (Taraka, Japan) was used to extract the plasmid according to the manufacturer’s instructions (Code No. 9760, Taraka). Finally, the concentration of the final cDNA library was detected.

### Yeast one-hybrid library screening

The bait fragment used for yeast one-hybrid library screening was designed based on the CArG element found in the wild-type *lc*. It consisted of three tandem copies of a specific nucleotide sequence, which is listed in the **Supplementary Table**. Two oligonucleotide chains of the bait fragment were synthesized using the gene-synthesis technique and mixed in a 1:1 ratio. The reaction program consisted of 95°C for 30 s, 72°C for 2 min, 37°C for 2 min, 25°C for 2 min, and storage at 4°C. To generate the bait vector, the bait sequence was cloned into the pAbAi vector, which was digested with *Xho*I and *Sma*I restriction enzymes. The resulting construct was named pAbAi-*lc*. Next, the bait vector pAbAi-*lc* was digested with *Bbs*I and *Bstb*I enzymes. The p53 vector was used as a positive control. The linearized pAbAi-*lc* and p53 vectors were then transformed into Y1HGold yeast-competent cells using Yeast Transformation System 2 (Taraka, Japan) according to the manufacturer’s instructions (Code No. 630439, Taraka). The transformed products were detected using Matchmaker Insert Check PCR Mix 1 (Taraka, Japan). The concentration of aureobasidin A (AbA) in the bait and positive control yeast strains was determined using Matchmaker Gold Yeast One-Hybrid Library Screening System Kit (Clontech, Japan, Mountain View, CA, USA) according to the manufacturer’s instructions (Code No. 630491, Clontech). For library screening, 15 μg of the cDNA library was transformed into Y1HGold[pAbAi-*lc*] yeast-competent cells. The resulting product was diluted 10-, 100-, and 10^3^-fold, respectively, and then cultured on SD/-Leu and SD/-Leu/AbA medium (90 mm). The number of colonies was counted on the 100-fold dilution medium, and the rest of the reaction liquid was cultured on SD/-Leu/AbA medium (150 mm). The number of colonies obtained was calculated using the formula: number of single colonies × dilution times × suspension volume (mL) ÷ volume on medium (mL). Finally, the colonies on the 150-mm medium were detected using Matchmaker Insert Check PCR Mix 2 (Taraka, Japan).

### Yeast one-hybrid assay

The coding sequence of *SlSEP3* was inserted into the pGADT7 vector (Clontech), while the CArG element in the wild-type *lc* was cloned into the pAbAi vector (Clontech) individually. The assay was performed with the Matchmaker Gold Yeast One-Hybrid Library Screening System Kit (Clontech, Japan) according to the manufacturer’s instructions (Code No.630491, Clontech). Primers used are listed in the **Supplementary Table**.

### Electrophoretic mobility shift assay

The coding sequence of *SlSEP3* was cloned into the pGADT7 vector and transformed into Y1HGold yeast-competent cells. Positive colonies were cultured in a YPDA liquid medium containing 50 mg mL^−1^ ampicillin at 30°C until the OD_600_ value reached 1.0. Yeast protein was extracted using a yeast protein extraction kit (Solarbio, Beijing, China) following the manufacturer’s instructions (Code No. BC3780, Solarbio). The extracted protein was purified using an Amicon^®^ Ultra-4 centrifugal filter (Merck, Burlington, MA, USA) to remove small molecule salts and heteroproteins according to the manufacturer’s instructions (Code No. UFC8010, Merck). The purified protein was dissolved in 1× PBS buffer and stored at −20°C in a refrigerator.

Electrophoretic mobility shift assay (EMSA) assay was performed using a Chemiluminescent EMSA Kit (Beyotime, Shanghai, China) according to the manufacturer’s instructions (Code No. GS009, Beyotime). The biotin-labeled probe was prepared by SBS Genetech Co. Ltd. (Beijing, China). All oligonucleotide probes used are listed in the **Supplementary Table**.

### Phylogenetic tree construction

Protein sequences of E-class genes involved in flower development in *Arabidopsis* and tomatoes were retrieved from the NCBI website. Multiple sequence alignment was conducted using Clustalw, and the neighbor-joining model was employed for constructing the phylogenetic tree.

### VIGS

SlSEP3 silencing fragments were designed using the virus induced gene silencing (VIGS) tool on the Sol Genomics Network (https://solgenomics.net/) and amplified by PCR, then ligated into the pTRV2 vector to generate the pTRV2-*SlSEP3* constructs. The pTRV2-*SlSEP3*, pTRV2 empty vector, and pTRV1 empty vectors were transformed into *Agrobacterium* (*Agrobacterium tumefaciens*) strain GV3101 individually. A mixture of cultures containing pTRV1 and pTRV2-*SlSEP3* was used for infection as previously described (Liu et al., 2002). Two infections were performed, one on germinated seeds and the other on stems. Silencing efficiency was determined using qRT-PCR on both young buds and leaves below the infected site. The primers used are listed in the **Supplementary Table**.

### Yeast two-hybrid assay

The coding sequence of the histone deacetylase *SlHDAs* was cloned into the pGADT7 vector (Clontech, Japan), while the coding sequence of *SlSEP3* was cloned into the pGBKT7 vector (Clontech, Japan). The assay was performed with the Matchmaker Gold Yeast Two-Hybrid System Kit (Clontech, Japan) according to the manufacturer’s instructions (Code No. 630489, Clontech, Japan). Primers used are listed in the **Supplementary Table**.

## Results

### *lc* mutant increased the number of floral organs and fruit locules and affected the *SlWUS* expression in tomatoes

**Figure 1:**
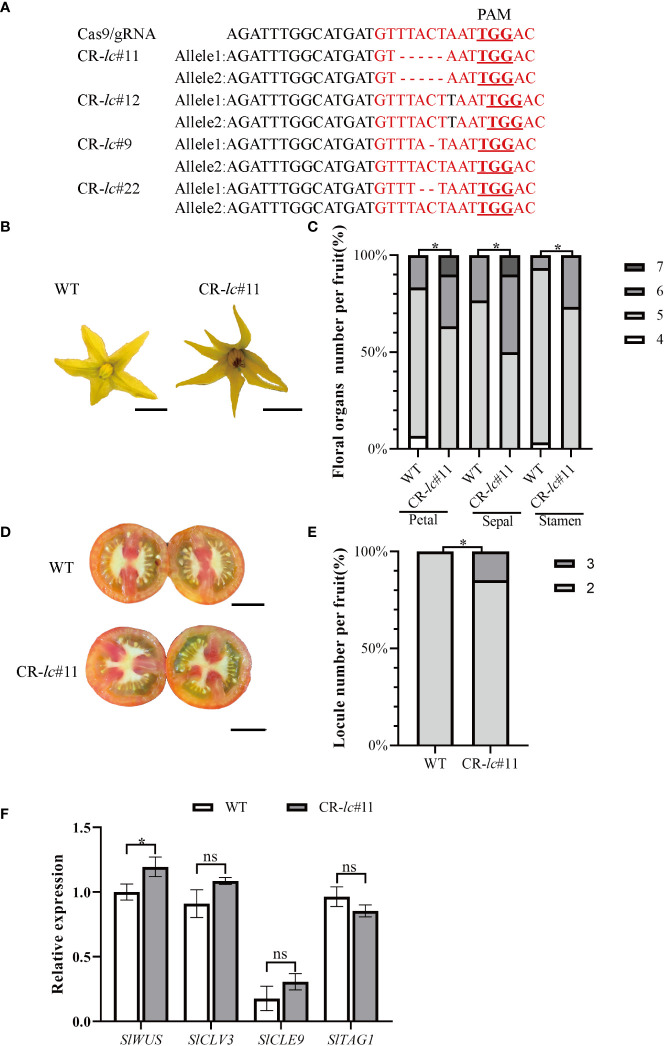
Tomato *lc* mutation increases the number of floral organs and fruit locules by altering the expression of the *SlWUS*. **(A)** Identification of CArG-motif (red font) editing in tomato *lc* ^CR^ mutants: the red font with underline indicates PAM sequences. **(B)** Comparison of flower phenotypes between the *lc* ^CR^ mutant and WT. Bars represent 0.5 cm. **(C)** Percentage of floral organ count statistics between the *lc* ^CR^ mutant and WT. **(D)** Comparison of *lc* ^CR^ and WT fruit cross-sectional phenotypes. Bars represent 1.0 cm. **(E)** The percentage of single fruit locule number count statistics between the *lc* ^CR^ mutant and WT. **(F)** Analysis of gene expression related to meristematic differentiation in tomato *lc* ^CR^ T_1_ generation. The data are presented as means ± SD of three independent tests. Significant differences were determined using an unpaired two-tailed Student’s *t*-test: ^*^ *p*-value < 0.05; ns, no significant difference.

To investigate the molecular function of *lc* in affecting tomato locule number, four *lc* mutants (#11, #12, #9, and #22) were generated using the CRISPR/Cas9 system under AC background. The *lc* target region in *T*_1_ generation plants was amplified and sequenced, revealing a 5-bp deletion, 1-bp insertion, 1-bp deletion, and 2-bp deletion in the CArG motif of the target sequences of *T*_1_ generation lines (**Figure 1A**). Compared with WT, the homozygous CR-*lc*#11 mutant showed an increase in floral organ number (**Figures 1B, C**; **Supplemental Dataset**), with some partial flowers composed of five to seven sepals, five to six stamens, and five to seven petals (**Figure 1C**). Additionally, the locule number increased from two to three (**Figure 1D**), with 14.2% of the fruits from the CR-*lc*#11 mutant exhibiting three locules (**Figure 1E**; **Supplemental Dataset**). However, most flowers in the CR-*lc*#11 mutant remained unchanged from the WT, with flowers composed of four to five sepals, four to five stamens, and four to five petals. In addition, we also analyzed the phenotype of flower organ number and locule number in homozygous CR-*lc*#12 mutant (**Supplementary Figure S1**, **Supplemental Dataset**). Similar to the CR-*lc*#11 mutant, the number of petals, sepals, and stamens increased compared with WT in the CR-*lc*#12 mutant. Furthermore, 10.7% of the fruits exhibited three locules. These findings suggest that the *lc* mutation affects the differentiation of stem cells in partial flowers.

In the CR-*lc*#11 mutant, we found that *SlWUS* expression was significantly increased during the differentiation of stem cells, while the expression of *SlCLV3*, *SlTAG1*, and *SlCLE9* was not changed (**Figure 1F**; **Supplemental Dataset**). It suggested that *lc* might change locule number by affecting *SlWUS* transcription.

### Construction of a tomato shoot apical cDNA library and screening test of MADS-box transcription factor SlSEP3 binding to *lc*

To investigate the upstream regulation of tomato *lc*, we first constructed a shoot apical cDNA library for screening proteins that bind to *lc*. We extracted shoot apical RNA from different stages of flower bud differentiation in tomato AC (**Supplementary Figure S2A**) and synthesized double-stranded cDNA from equal amounts of total RNA. The resulting electrophoretic bands of double-stranded cDNA fragments were dispersed, with a majority concentrated in the 500- to 3,000-bp range (**Supplementary Figure S2B**). We purified the cDNA by removing highly expressed components and retaining the effective fragments within the enriched interval (**Supplementary Figure S2C**). The normalized full-length cDNA was then digested with *Sfi*I (**Supplementary Figure S2D**) and ligated to a three-frame pGADT7-*Sfi*I vector to generate the primary cDNA library. We ultimately transformed the primary cDNA library into competent cells from *Escherichia coli* and measured its capacity, which was calculated based on the number of colonies. The primary cDNA library capacity of the three reading frame vectors was 2.6 × 10^6^ cfu, 1.6 × 10^6^ cfu, and 2.4 × 10^6^ cfu, respectively. Finally, we detected the length of inserted fragments, which was mainly distributed in the 500- to 3,000-bp range (**Supplementary Figure S2E**). We mixed the colonies for germiculture in an LB liquid medium, the concentration of the final cDNA library plasmid was 1 mg mL^−1^.

Secondly, we performed a yeast one-hybrid screen. We selected an OD_600_ value of 0.002 to screen for the appropriate concentration of AbA (**Supplementary Figure S3A**). We found that 150 ng mL^−1^ of AbA completely inhibited yeast growth. To ensure the reliability of subsequent library screens, we selected a concentration of 200 ng mL^−1^ of AbA. We transformed the final cDNA library into a bait yeast strain and used the P53 positive yeast strain as a control. The number of positive control colonies on SD/-Leu and SD/-Leu/AbA^200^ agar mediums was roughly the same, but the number of bait yeast strain colonies on SD/-Leu/AbA^200^ agar medium was significantly lower than that on SD/-Leu agar medium. We counted 137 single colonies on SD/-Leu/AbA^200^ agar medium (**Supplementary Figure S3B**). The number of obtained clones was calculated to be 137 cfu/0.1 mL × 100 mL × 15 mL = 2.055 × 10^6^, which was greater than 10^6^, indicating that the library screen test was successful.

Finally, we selected every single colony on the 150-mm agar medium for PCR detection and sequencing. The obtained results were analyzed on the Tomato Genomics Network, and we obtained statistical information about the function of the screened factors (**Table 1**). Eventually, two candidate transcription factors were identified: Solyc05g015750, which encodes SEPALLATA3 (SEP3) of the tomato MADS-box transcription factor, and Solyc02g092370, which encodes GARS11 of the tomato GRAS family transcription factor. Based on the function of transcription factors, we considered SlSEP3 to be a potential candidate for binding to *lc*.

**Table 1:** Interaction factors bound with *lc* screened by yeast one-hybrid.

Order	Locus	Description
1	*Solyc02g038814*	Unknown protein
2	*Solyc01g111600*	Metal ion-binding protein
3	*Solyc05g015750*	SEP3 MADS-box transcription factor
4	*Solyc12g089030*	RUB1-conjugating enzyme
5	*Solyc04g080240*	PERQ amino acid-rich with GYF domain-containing protein 2
6	*Solyc01g095410*	Eukaryotic translation initiation factor 1A
7	*Solyc04g082840*	B2-type cyclin-dependent kinase cdkb2
8	*Solyc02g092370*	GRAS family transcription factor
9	*Solyc05g056470*	ABC transporter G family member 5
10	*Solyc10g008190*	OB-fold nucleic acid-binding domain-containing protein
11	*Solyc09g075150*	60S ribosomal protein L22-2
12	*Solyc01g099840*	Auxin-repressed protein
13	*Solyc05g014640*	En/Spm-like transposon protein
14	*Solyc04g079970*	rce1
15	*Solyc01g104080*	Hypothetical membrane-spanning protein
16	*Solyc01g103450*	Chaperone DnaK
17	*Solyc09g074300*	Histone H2A
18	*Solyc05g012440*	Cytochrome c oxidase assembly protein ctaG
19	*Solyc09g065330*	40S ribosomal protein S24
20	*Solyc06g053620*	Phosphoenolpyruvate carboxylase kinase 2
21	*Solyc01g099340*	Zinc finger protein
22	*Solyc11g072240*	*SlCaM4* is a member of the calmodulin gene family, which modulates the response to calcium signaling
23	*Solyc03g121060*	*SlIAA26/IAA26* is a member of the Aux/IAA gene family of tomato

**Figure 2:**
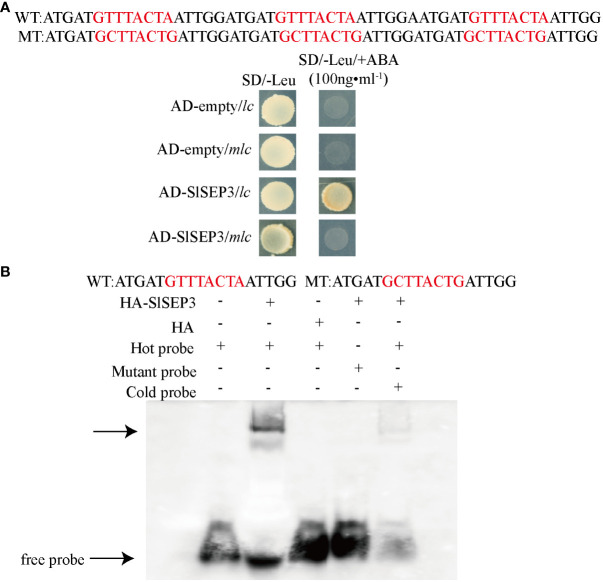
*In vivo* yeast one-hybrid **(A)** and *in vitro* EMSA validation **(B)** of the SlSEP3 transcription factor bound to the wild-type *lc*.

To ensure the reliability of yeast one-hybrid screening results, we implemented a yeast one-hybrid assay and an electrophoretic mobility shift assay. We recombined the coding region of *SlSEP3* with the pGADT7 vector and performed a yeast one-hybrid assay with the CArG element in wild-type *lc*. We used an empty pGADT7 vector as the negative control and mutant *lc* as a control (**Figure 2A**). The results demonstrated that the SlSEP3 transcription factor interacted with the wild-type *lc* but not with the mutant *lc*. Furthermore, we transferred the recombinant expression vector pGADT7-SlSEP3 into yeast Y1HGold-competent cells for cultivation and obtained a purified recombinant HA-tagged SlSEP3 protein from yeast for the EMSA. To exclude any influence from HA-tagged yeast protein, it was set as the experimental control. We confirmed that HA-SlSEP3 yeast protein bound to the wild-type *lc*, and the binding ability decreased when the concentration of the cold competition probe was increased to 200 times (**Figure 2B**). Taken together, the results from the yeast one-hybrid assay and EMSA provided strong evidence supporting the reliability of the yeast one-hybrid screening.

### Tomato SlSEP3 affected the *SlWUS* expression and locule number

**Figure 3:**
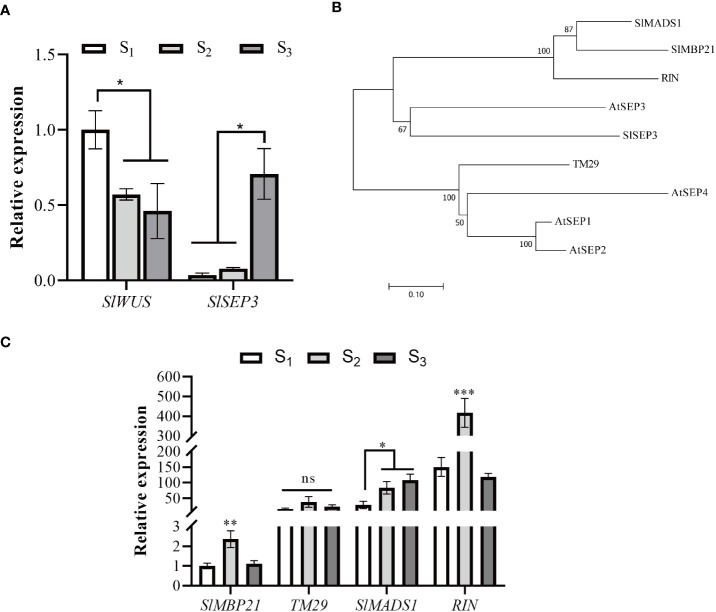
Tomato SlSEP3 and *SlWUS* have opposite effects on flower bud differentiation **(A)**. Expression analysis of *SlSEP3* and *SlWUS* in tomato flower bud differentiation stages. S_1_, S_2_, and S_3_ respectively represent early flower bud differentiation, middle flower bud differentiation (sepal and petal formation stage), and late flower bud differentiation stages (carpel formation stage). The data are presented as the means ± SD of three independent tests. Significant differences were determined using one-way ANOVA with Dunnett’s test: ^*^ *p*-value < 0.05. **(B)** Systematic phylogenetic analysis of E-class genes involved in tomato and *Arabidopsis* flower development. The evolutionary history was inferred using the neighbor-joining method. **(C)** Expression analysis of E-class genes involved in tomato flower bud differentiation stages. The data are presented as the means ± SD of three independent tests. Significant differences were determined using one-way ANOVA with Tukey’s multiple comparisons test: ^*^ *p*-value < 0.05; ^**^ *p*-value < 0.01; ^***^ *p*-value < 0.0005.

In order to elucidate the role of *SlSEP3* in regulating tomato locule number, we performed qRT-PCR analysis to examine the expression level of *SlSEP3* and *SlWUS* in the shoot apical (**Figure 3**). Our findings revealed that *SlWUS* expression was downregulated during flower bud differentiation, particularly after the early stage, whereas the expression of *SlSEP3* was upregulated, especially after the sepal and petal formation. Notably, the expression patterns of *SlSEP3* and *SlWUS* were found to be opposite to each other. Given that SlSEP3 functions as a transcription factor that interacts with *lc*, it is plausible to speculate that SlSEP3 may serve as an inhibitor of *SlWUS*. In addition, we performed a phylogenetic analysis of the E-class genes that influence tomato flower development. There are five E-class genes in tomato, and upon comparing them with the *AtSEP* in *Arabidopsis*, it was observed that tomato *SlSEP3* clustered preferentially with *AtSEP3*. This suggests that tomato *SlSEP3* shares a similar function with *AtSEP3* in *Arabidopsis*. By analyzing the expression patterns of other E-class genes at different stages of floral bud differentiation in wild-type tomatoes, we found that the *SlMADS1* and *SlSEP3* might have similar expression patterns. However, further research is needed to determine whether there is functional redundancy between these two genes in regulating fruit locule number.

**Figure 4:**
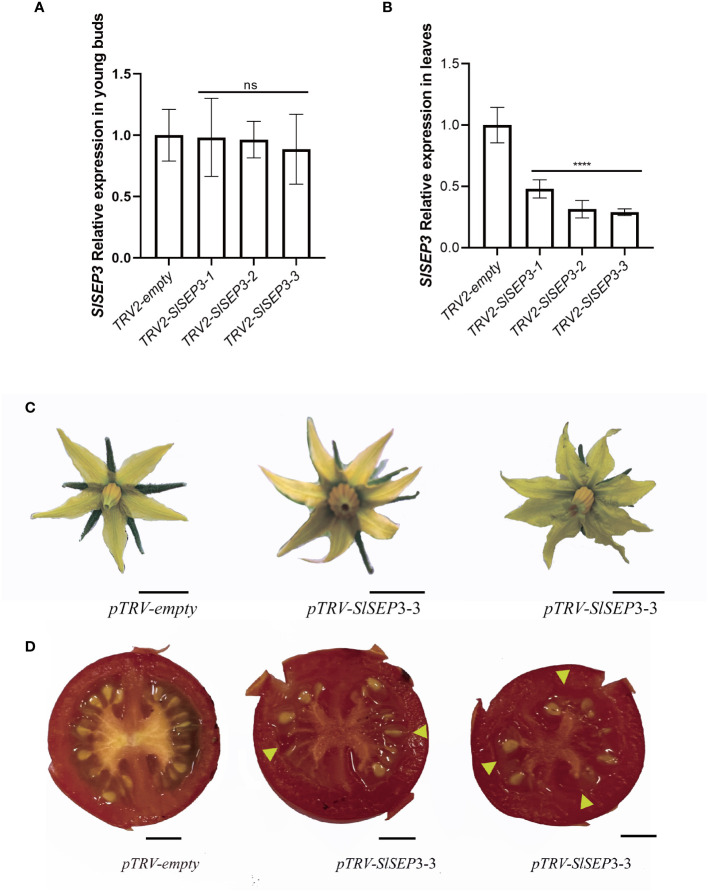
The number of petals and locules in partially silenced *SlSEP3* plants increased. **(A)** Detection of the transient silencing efficiency of *SlSEP3* in young buds. **(B)** Detection of the transient silencing efficiency of *SlSEP3* in leaves. Data are the means ± SD of three independent tests. Significant differences were performed with a two-way ANOVA with Dunnett’s multiple comparisons test: ^****^ *p*-value < 0.0001; ns, no significant difference. **(C, D)** Comparison of flower phenotypes **(C)** and fruit cross-sectional phenotypes **(D)** between the SlSEP3-3-silenced plant line and the control plant line. Bars represent 0.5 cm.

**Figure 5:**
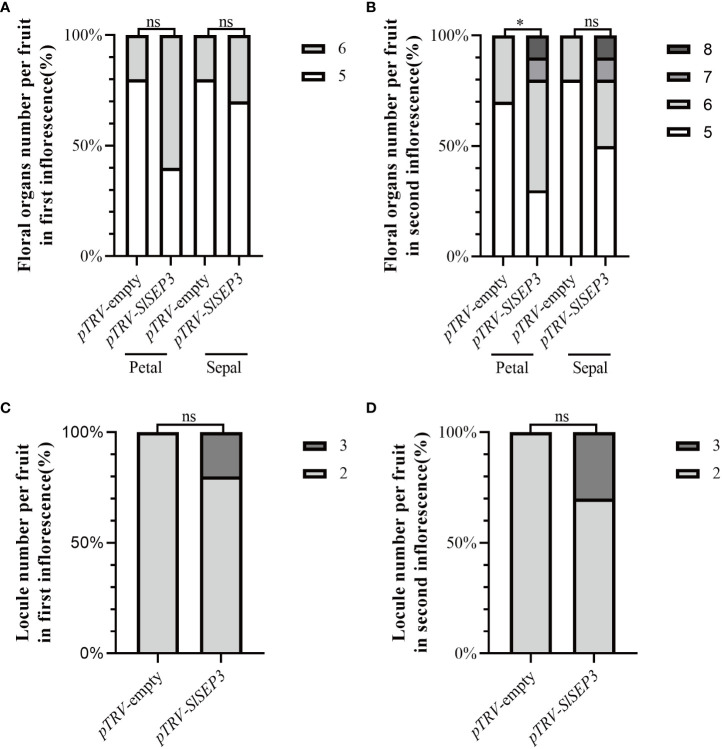
Silencing of *SlSEP3* results in an increase in the number of floral organs and fruit locules. Percentage of the floral organ **(A, B)** count statistics and single fruit locule number **(C, D)** count statistics between the SlSEP3-silenced plants and the control plant line. Significant differences were determined using a two-way ANOVA with Sidak’s multiple comparisons test: ^*^ *p*-value < 0.05; ns, no significant difference.

To further investigate whether SlSEP3 is a negative regulator of fruit locule number, we generated a set of tomato lines with individually silenced *SlSEP3* using virus-induced gene silencing and analyzed *SlSEP3* expression in young buds and leaves by qRT-PCR (**Figures 4A, B**; **Supplemental Dataset**). Compared with wild-type plants, pTRV-*SlSEP3* plants displayed an increase in the number of floral organs in both the first and second inflorescence (**Figures 4C**, **5A, B**; **Supplemental Dataset**). In the first inflorescence, only 20% of the flowers in wild-type plants had six petals and six sepals, whereas 60% of flowers in pTRV-*SlSEP3* plants had six petals and 30% of the flowers had six sepals (**Figure 5A**). Moreover, the number of petals in the second inflorescence of pTRV-*SlSEP3* plants was significantly increased (**Figure 5B**), particularly in pTRV-*SlSEP3*-3 plants where seven and eight petals were observed (**Figure 4C**). Furthermore, there was an increase in the number of locules in both the first and second inflorescence of pTRV-*SlSEP3* plants, with 20% of the fruits in the first inflorescence and 30% of the fruits in the second inflorescence of pTRV-*SlSEP3* plants having three locules (**Figures 4D**, **5C, D**). These results suggested that *SlSEP3* acts as a negative regulator of tomato locule number.

### Tomato SlSEP3 interacts with histone deacetylase SlHDAs

**Figure 6:**
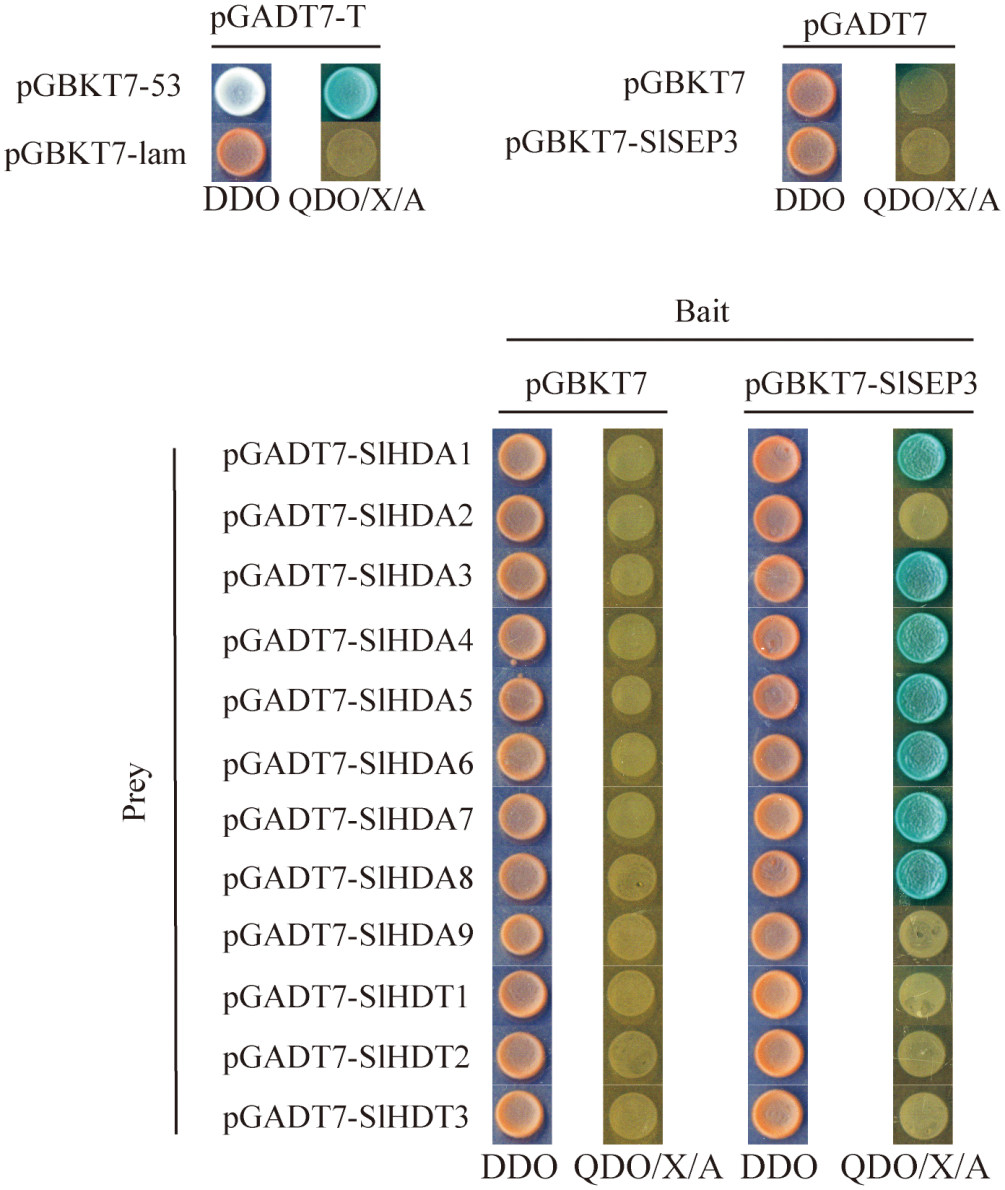
Yeast two-hybrid analysis of the interaction between tomato SlSEP3 transcription factor and the histone deacetylase family.

To further investigate the regulation of *SlWUS* expression by the tomato SlSEP3 transcription factor after binding to *lc*, we conducted an analysis of the interaction between SlSEP3 and tomato histone deacetylase, drawing upon the functions of homologous genes in *Arabidopsis* (**Figure 6**). We cloned the coding sequences of SlHDA1–9 and SlHDT1–3 from the tomato histone deacetylase family and performed yeast two-hybrid assays. The results provide preliminary evidence that SlSEP3 could interact with several tomato histone deacetylases, including SlHDA1, SlHDA3, SlHDA4, SlHDA5, SlHDA6, SlHDA7, and SlHDA8 in yeast cells.

## Discussion

In wild-type tomatoes, the CArG-motif of *lc* is mainly composed of “GTTTACTAATTGGAC”. However, in the *lc* mutant, there is a mutation at the second and eighth nucleotides. This mutation has been shown to increase the fruit locule number to three to four (Barrero et al., 2006; Muños et al., 2011). In this study, we deleted a 5-bp fragment of the CArG motif in the *lc* ^CR^ under AC background and found that 14.2% of fruits had locule numbers up to 3 (**Figures 1A–E**; **Supplemental Dataset**). This is consistent with the phenotype of 11% fruits in *S.pim*-*lc* ^NIL^ with three to four locules (Rodríguez-Leal et al., 2017). Additionally, we also observed a slight increase in floral organ number and fruit locule number in the *lc* ^CR^ with a 1-bp insertion in the CArG-motif, with 10.7% of fruits displaying three locules (**Supplementary Figure S1**, **Supplemental Dataset**). Furthermore, since the *lc* does not encode any protein, we are particularly interested in obtaining strains with a complete deletion of the cArG motif. Considering that the CR-*lc*#12 strain only exhibited a 1-bp insertion at the target site, we believe that the CR-*lc* #11 strain is more suitable. These results suggest that the CArG motif in *lc* is involved in the differentiation of stem cells during floral development. Previous studies have shown that *SlWUS* is upregulated after natural variation in *lc* and may be the target of *lc* (Li et al., 2017). In the CR-*lc*#11 mutant, only *SlWUS* expression was significantly increased, while other genes related to stem cell differentiation, such as *SlCLV3*, *SlTAG1*, and *SlCLE9*, were unaffected (**Figure 1F**, **Supplemental Dataset**). This suggests that the *lc* site may directly affect *SlWUS* transcription to change locule number, although the specific mechanism remains unclear. It is worth noting that although the upregulation of *SlWUS* expression reaches a significant level, the magnitude of change is only around 20%. Therefore, in order to accurately clarify the tissue-specific *SlWUS* expression in *lc* ^CR^ mutants, we plan to utilize transgenic plants with a *SlWUS* promoter fused with GFP or GUS tags for further in-depth analysis in our future studies.

In *Arabidopsis*, the MADS-box transcription factor *AtSEP* is involved in floral organ formation and the establishment of whorled organ patterns (Theissen, 2001; Becker and Theissen, 2003). Loss-of-function mutations in the *sep3* gene lead to a reduced number and size of floral organs, resulting in defective flower development with no petals, stamens, or carpels. These phenotypes are similar to those observed in *pin1* and *arf3* (Kaufmann et al., 2009). To identify potential regulators of the *lc* site, a Y1H library screening was performed, which identified 23 proteins that bind to the *lc* and activate GAL4 transcription (**Table 1**). Among these proteins, a MADS-box transcription factor known as SlSEP3 (Solyc05g015750) was found. The Y1H and EMSA results confirmed the binding of SlSEP3 to the *lc* site (**Figures 2A, B**). This suggests that *SlSEP3* may be involved in regulating *SlWUS* expression. To further investigate the function of *SlSEP3*, VIGS was used to silence *SlSEP3* expression. The floral organ and fruit phenotype in *SlSEP3*-silenced plants resembled those of the *lc* mutant (**Figures 1B, D**, **4C, D**). Additionally, the number of sepals, stamens, and petals were similarly affected in *lc* mutant and *SlSEP3*-silenced plants (**Figures 1C, E**, **5A, B**). Moreover, *SlSEP3* expression was up-regulated during flower bud differentiation, particularly in the carpel formation stage (**Figure 3A**). These results suggest that SlSEP3 affects locule formation by binding to the *lc* site. However, due to the limitations of the VIGS method, it is important to obtain CRISPR-edited SlSEP3 mutants to better elucidate the role of *SlSEP3* in tomato locules. Additionally, as the *lc* site is located after the 3′UTR of *SlWUS* in the genome, the mechanism of its function remains unclear.

Studies have indicated that AtSEP3 can activate the expression of class B and C genes by recruiting BRM and SYD to their promoter regions, thus overcoming PRC2-mediated inhibition (Wu et al., 2012). AtSEP3 can also form tetramers with AG to inhibit H3K27me3 and activate the expression of *CRC* and *KNU* (Hugouvieux et al., 2018). AG tetramers bind to Polycomb response elements in *KNU* promoter and compete for binding to histone H3, ultimately ensuring the normal differentiation of meristem by inhibiting *WUS* expression (Sun et al., 2014). In tomatoes, MADS-box transcription factors TAG1 and tomato MADS-box 29 (TM29) jointly regulate reproductive development by recruiting histone deacetylases PRD3/HDA1 subfamily genes *SlHDA1* and *SlHDA4*. *TM29* is highly expressed in the primordium of all four-wheel floral organs, and downregulated expression of *TM29* leads to parthenogenesis and abnormalities in the second and third-round floral organs (Ampomah-Dwamena et al., 2002). In this study, it was found that SlSEP3 could recruit some PRD3/HDA1 subfamily members such as SlHDA1, SlHDA3, SlHDA4, SlHDA5, SlHDA6, SlHDA7, and SlHDA8 (**Figure 6**). However, a BIFC or pull-down assay is needed to further investigate the interaction between SlHDA and SlSEP3. Additionally, the increase in *SlSEP3* expression also promoted the recruitment of SlHDA proteins near the *lc* site during flower bud differentiation (**Figure 3**). This suggests that the nucleosome near the *lc* site may not be easily opened, leading to the inhibition of transcription of nearby genes. Moreover, the *lc* site is located after the 3’UTR of *SlWUS* in the genome, so the inhibitory effect on *SlWUS* transcription is relatively weak. This also explains why only some fruits have an increase in locule number after *lc* mutation and *SlSEP3* silencing.

**Figure 7:**
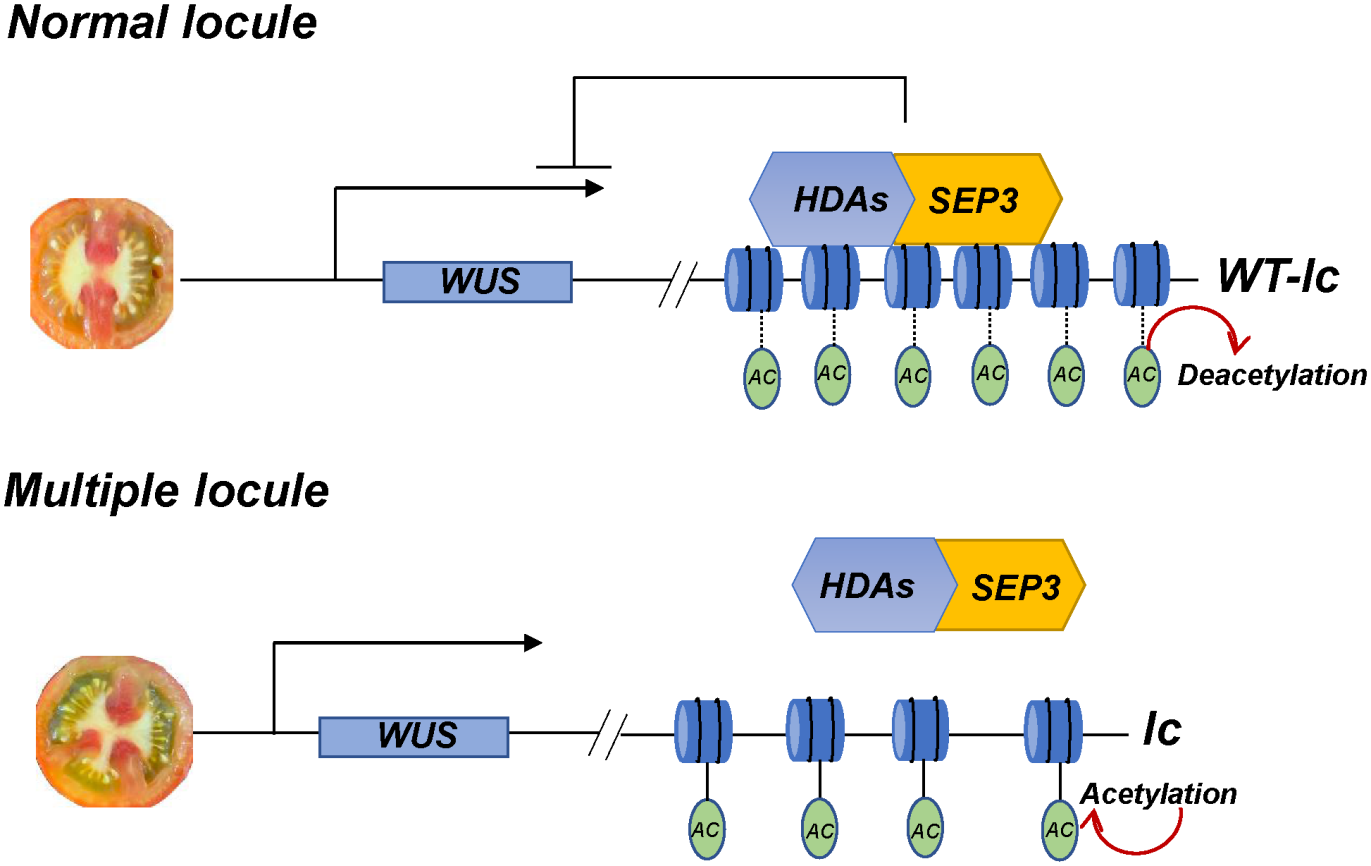
The module of *lc* QTL is responsible for regulating tomato locule formation. In normal locule tomato plants, the MADS-box transcription factor SlSEP3 binds to *lc* and recruits histone deacetylase SlHDAs. This process removes the charge and acetyl (Ac) on histone lysine residues, resulting in DNA tightly binding to histone nucleosomes, which limits the effects of transcription factors, polymerase, and DNA. Consequently, the transcription of *SlWUS* near *lc* is inhibited, and the locule number is maintained at a normal level. In multiple locule tomato plants, the relevant inhibitory effect is relieved, and the chromatin nucleosome structure will relax, maintaining normal transcription of *SlWUS* and resulting in an increase in the locule number.

In conclusion, the *lc* site has a significant impact on the development of tomato locules, and its mutation can increase the number of locules in the fruit. The SlSEP3 protein initially binds to the *lc* site and then recruits several histone deacetylases, including SlHDA, to inhibit nucleosome relaxation. This ultimately affects the transcription of *SlWUS*, which regulates the locule number in the fruit (**Figure 7**). Therefore, the *SlSEP3*-*SlHDA*-*lc*-*SlWUS* module is a crucial addition to enhancing the molecular network of tomato locule formation.

## Data availability statement

The original contributions presented in the study are included in the article/**Supplementary Material**. Further inquiries can be directed to the corresponding authors.

## Author contributions

HX and SM designed and drafted the manuscript. HX and SM conducted experiments and data analysis. YY and LH participated in some data analysis. JM and CT conducted a field data survey. YH and YC help review and edit manuscripts. MQ and TL were involved in the critical revision of important intellectual content and project management. All authors contributed to the manuscript revision and read and approved the submitted version.
