# Supplementary material for: A molecular framework for lc controlled locule development of the floral meristem in tomato
Source: Front Plant Sci. 2023 Aug 23;14:1249760. doi: 10.3389/fpls.2023.1249760 (PMC10482247; doi:10.3389/fpls.2023.1249760)
Supplement: Supplementary file 1 [file DataSheet_1.docx]

# A molecular framework for *lc* controlled locule development of the floral meristem in tomato

Hengzuo Xiang ^1,2,3,4,5†^, Sida Meng ^1,2,3,4,5†^, Yunzhu Ye^1,2,3,4,5^, Leilei Han^1,2,3,4,5^, Yi He^2,3^, Yiqing Cui^2,3^, Changhua Tan^1,2,3,4,5^, Jian Ma^1,2,3,4,5^ Mingfang Qi^1,2,3,4,5^* and Tianlai Li^1,2,3,4,5^*

*** Correspondence:**Mingfang Qi:[qimingfang@syau.edu.cn](mailto:qimingfang@syau.edu.cn)

Tianlai Li:ltl@syau.edu.cn


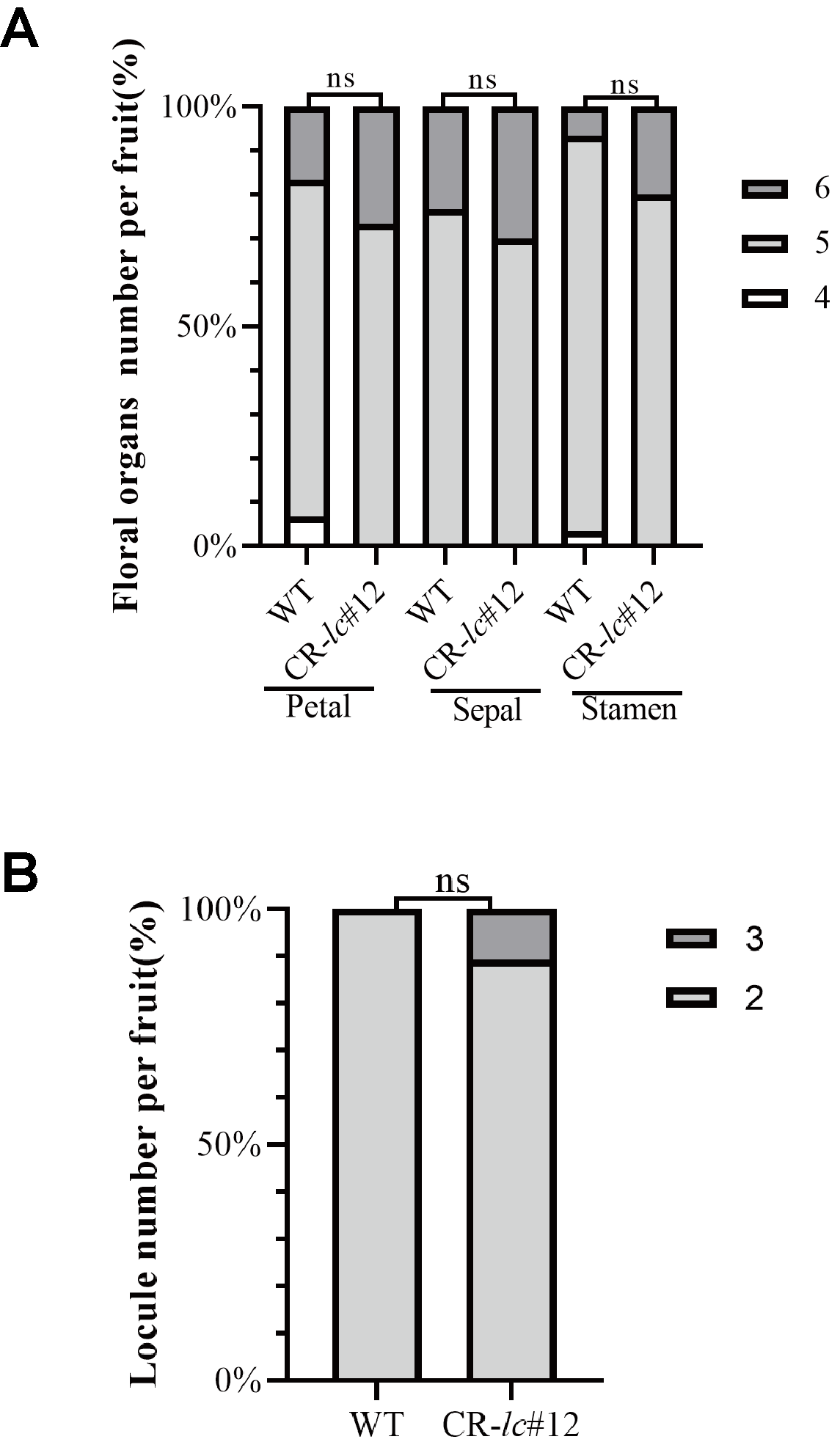


Figure S1. Percentage of floral organ number (**A**) and single fruit locule number **(B**) count statistics between CR-*lc*#12 mutant and WT. Significant differences were determined using an unpaired two-tailed Student’s t-test: ns, no significant difference.


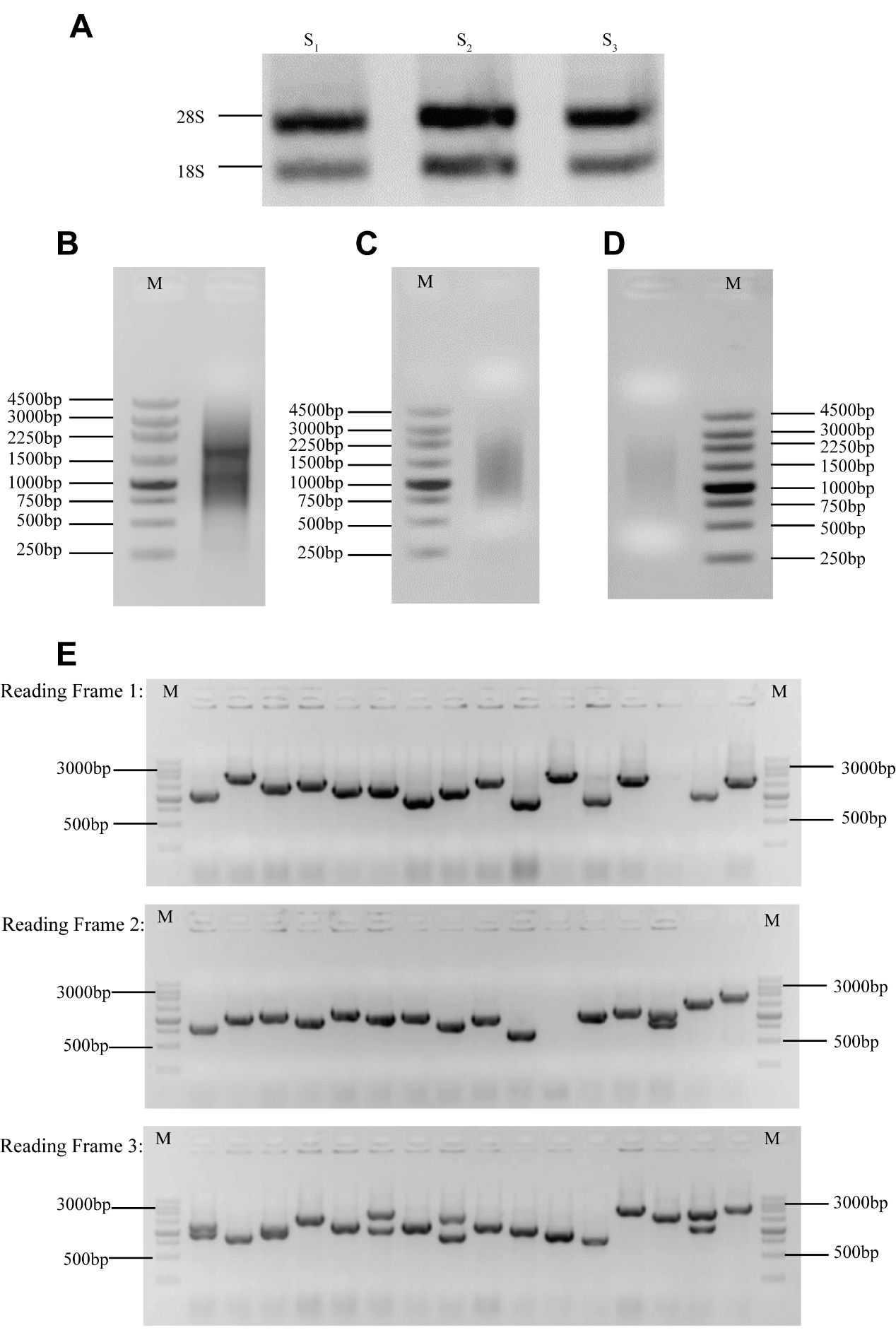


Figure S2. Construction of a cDNA library from the shoot apical meristem of tomato plants. (**A).** Total RNA at different flower bud differentiation stages in tomato AC. (**B**)**.** Purified double-stranded cDNA. (**C**)**.** Normalized full-length cDNA. (**D**)**.** Small fragment removed cDNA. (**E**)**.** Insert size detection from 48 randomly colonies of tomato shoot apical cDNA library.


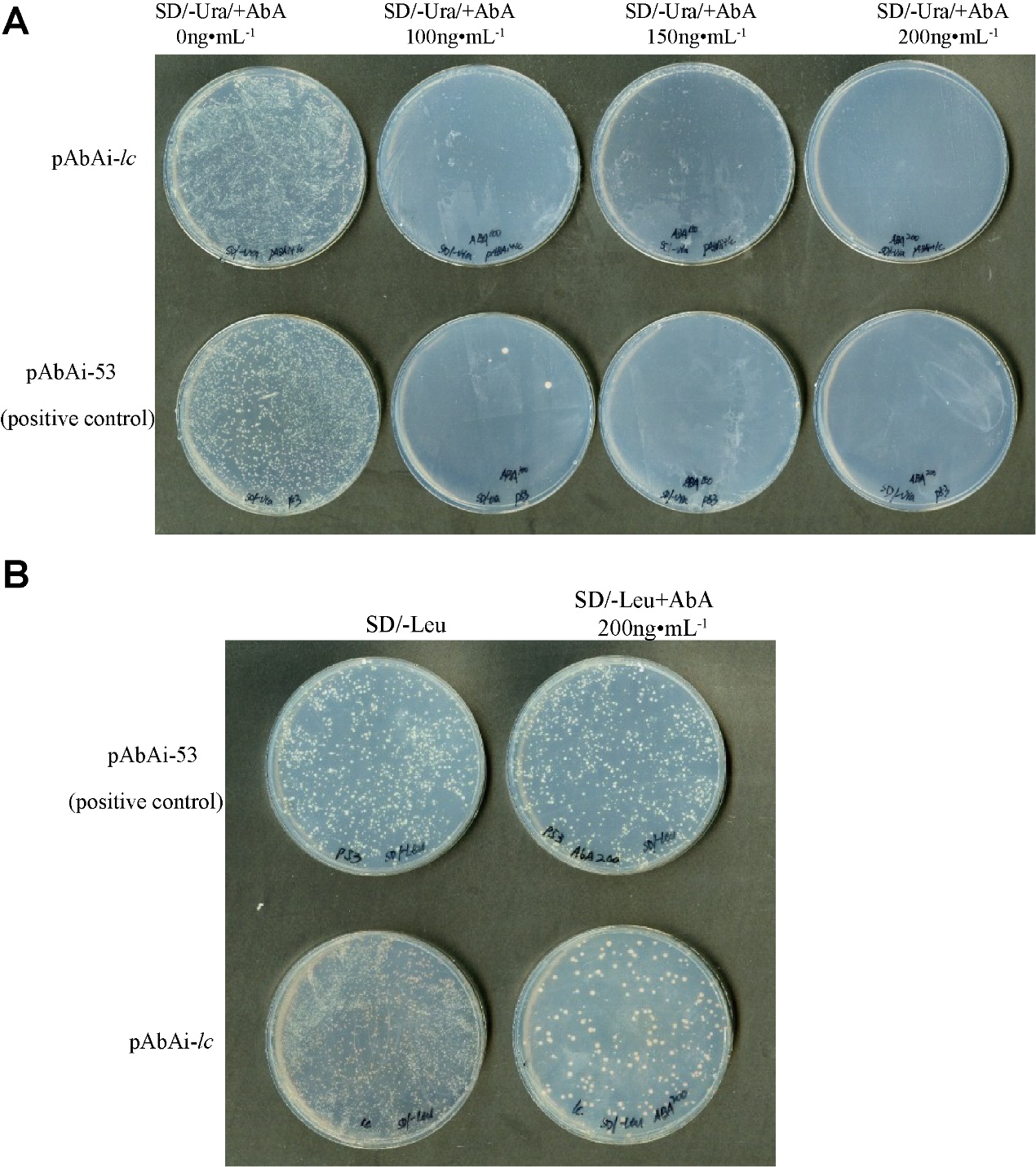


Figure S3. Screening of yeast one-hybrid library of tomato shoot apical. (**A).**Growth of yeast bait strains Y1HGold[pAbAi-*lc*] and positive control with different concentrations of ABA. (**B**)**.** Screening of yeast one-hybrid library of tomato shoot apical on SD/-Leu and SD/-Leu/AbA^200^ agar medium after 1% dilution of the library conversion solution.

Supplemental Table1. Primers used in this study

| Gene ID | Name | Sequences(5’-3’) | Application |
| --- | --- | --- | --- |
|  | *lc*-F | TATCTTCTGAACCACACGTCTCG | Mutant identification |
|  | *lc*-R | АТСССТСТАТССGТTАTCACCAT |  |
| Solyc02g083950 | *SlWUS*-F | CTGCTGCCTCTGCCACTGAT | qRT-PCR |
|  | *SlWUS*-R | ATGGACACTGAACACCTGGATTA |  |
| Solyc11g071380 | *SlCLV3*-F | ACAGAAAGGTTTTAGAAAGGCAGT | qRT-PCR |
|  | *SlCLV3*-R | GGAGTCTTAGGTTTCTTAGGACTAGC |  |
| Solyc02g071730 | *SlTAG1*-F | GCAGAAGAGGGAAGTTGATTTACA | qRT-PCR |
|  | *SlTAG1*-R | TGGAGGCACAAGCTCATGATA |  |
| Solyc06g074060 | *SlCLE9*-F | TGCAAGCACAATCCTCTGATC | qRT-PCR |
|  | *SlCLE9*-R | CATTATATTCCTGGGGAGACCTC |  |
| NM_001330119.1 | *ACTIN*-F | TGTCCCTATTTACGAGGGTTATGC | qRT-PCR |
|  | *ACTIN*-R | AGTTAAATCACGACCAGCAAGAT |  |
| Solyc05g015750 | *SlSEP3*-CDS-F | ATGGGAAGGGGTAGGGTTGAGCT | CDS amplification |
|  | *SlSEP3*-CDS-R | TCAAGGCAACCAGCCAGCCAT |  |
| Solyc05g015750 | *SlSEP3*-pTRV2-F | GTGAGTAAGGTTACCGAATTCATGGGAAGGGGTAGGGTTGA | VIGS |
|  | *SlSPE3*-pTRV2-R | CGTGAGCTCGGTACCGGATCCTTTAAGCTTCAAGTACTCCTGCTGG |  |
| Solyc05g015750 | *SlSEP3*-AD-F | GCCATGGAGGCCAGTGAATTCATGGGAAGGGGTAGGGTTGAGCT | Y2H |
|  | *SlSEP3*-AD-R | AGCTCGAGCTCGATGGATCCCTCAAGGCAACCAGCCAGCCAT |  |
| Solyc09g091440 | *SlHDA1*-AD-F | GCCATGGAGGCCAGTGAATTCATGGATGTTGGAGGAAACTCC | Y2H |
|  | *SlHDA1*-AD-R | AGCTCGAGCTCGATGGATCCCTTAGGAGATAATATCAGTTGGTTGATC |  |
| Solyc03g112410 | *SlHDA2*-AD-F | GCCATGGAGGCCAGTGAATTCATGGATTCTTCAGTCGTGGAG | Y2H |
|  | *SlHDA2*-AD-R | AGCTCGAGCTCGATGGATCCCTTAAAGATTGTAGTAATTACTGAACTTTC |  |
| Solyc06g071680 | *SlHDA3*-AD-F | GCCATGGAGGCCAGTGAATTCATGGACTCCTCCACCGTAGACG | Y2H |
|  | *SlHDA3*-AD-R | AGCTCGAGCTCGATGGATCCCCTAGGGATGATCATCAACCATGTC |  |
| Solyc11g067020 | *SlHDA4*-AD-F | GCCATGGAGGCCAGTGAATTCATGAGGTCCAAGGACAAAATCT | Y2H |
|  | *SlHDA4*-AD-R | AGCTCGAGCTCGATGGATCCCTTAGGCATCATCAGTGTGGTTAT |  |
| Solyc08g065350 | *SlHDA5*-AD-F | GCCATGGAGGCCAGTGAATTCATGTCATCCGCTGCCTCTTC | Y2H |
|  | *SlHDA5*-AD-R | AGCTCGAGCTCGATGGATCCCCTAGCTCTTCATATCTATCAGGGATTT |  |
| Solyc06g074080 | *SlHDA6*-AD-F | GCCATGGAGGCCAGTGAATTCATGGCTTCTTCAGCATCACAAT | Y2H |
|  | *SlHDA6*-AD-R | AGCTCGAGCTCGATGGATCCCCTAAGCATCTCTAAGAAAAGGTACAAC |  |
| Solyc01g009110 | *SlHDA7*-AD-F | GCCATGGAGGCCAGTGAATTCATGCAGACATTCCAAGAGTCG | Y2H |
|  | *SlHDA7*-AD-R | AGCTCGAGCTCGATGGATCCCTCAAAAGGAATGTATGTGCTTCAC |  |
| Solyc03g119730 | *SlHDA8*-AD-F | GCCATGGAGGCCAGTGAATTCATGATCTTAGTGCAAAAATGTGTT | Y2H |
|  | *SlHDA8*-AD-R | AGCTCGAGCTCGATGGATCCCTCAACAAGAAAAATTACAAGAGGAC |  |
| Solyc03g115150 | *SlHDA9*-AD-F | GCCATGGAGGCCAGTGAATTCATGGATTCCGGTGAACGACGG | Y2H |
|  | *SlHDA9*-AD-R | AGCTCGAGCTCGATGGATCCCTTACAATGCTCTGCTAGCTGCTTCTTG |  |
| Solyc09g009030 | *SlHDT1*-AD-F | GCCATGGAGGCCAGTGAATTCATGGAGTTTTGGGGTGCTGAG | Y2H |
|  | *SlHDT1*-AD-R | AGCTCGAGCTCGATGGATCCCTTACTTTCCAGCACTGTGTTTAGCT |  |
| Solyc10g085560 | *SlHDT2*-AD-F | GCCATGGAGGCCAGTGAATTCATGGAATTTTGGGGTGCGGAG | Y2H |
|  | *SlHDT2*-AD-R | AGCTCGAGCTCGATGGATCCCTTACTTTCCAGCGCTGTGCTTAGC |  |
| Solyc11g066840 | *SlHDT3*-AD-F | GCCATGGAGGCCAGTGAATTCATGGAGTTTTGGGGTGTGACAT | Y2H |
|  | *SlHDT3*-AD-R | AGCTCGAGCTCGATGGATCCCCTACTTCCTTCCTTGCTTGCC |  |
|  | F | ATGATGTTTACTAATTGG | EMSA |
|  | R | CCAATTAGTAAACATCAT |  |
|  | mF | ATGATGCTTACTGATTGG |  |
|  | mR | CCAATCAGTAAGCATCAT |  |
| Solyc05g015750 | *SlSPE3*-BK-F | ATGGCCATGGAGGCCGAATTCATGGGAAGGGGTAGGGTTGAGCT | Y2H |
|  | *SlSPE3*-BK-R | TAGTTATGCGGCCGCTGCAGGTCAAGGCAACCAGCCAGCCAT |  |
| Solyc05g015750 | *SlSPE3*-F | TTCTTTGTGATGCTGAGGTTG | qRT-PCR |
|  | *SlSPE3*-R | ATTTCCAGTGCTTCTCGTGTT |  |
| Solyc02g089200 | *SlTM29*-F | CAGCAGCAACATCCTCAATCTC | qRT-PCR |
|  | *SlTM29*-R | CACAGCATCCAACCAGGTATCA |  |
| Solyc03g114840 | *SlMADS1*-F | TGTGATGCTGAAGTTGCCCT | qRT-PCR |
|  | *SlMADS1*-R | AAAGTTTCTCTGAGATCGTTGGAG |  |
| Solyc05g012020 | *RIN*-F | ACCCAAACTTCATCAGATTCACA | qRT-PCR |
|  | *RIN*-R | TCCTCACCTAGCAAATGCCTT |  |
| Solyc12g038510 | *SlMBP21*-F | ATCTTTGGACCCGATGTTACC | qRT-PCR |
|  | *SlMBP21*-R | CCAGCATAGATTGAGTCTTTTTTG |  |
|  | Bait fragment | TCGAGCTCGGTACCCATGATGTTTACTAATTGGATGATGTTTACTAATTGGATGATGTTTACTAATTGGTCGAGGCATGTGCTC | Y1H screen |
|  | sgRNA | GTGGCATGATGTTTACTAAT | CRISPR-Cas9 |
